# Towards an interpretable deep learning model of anticancer drug sensitivity based on multi-omics data and multi-branch frameworks

**DOI:** 10.1093/bib/bbaf631.019

**Published:** 2025-12-12

**Authors:** Wei ding, Yang wu, Ming chen, Yufang qin

**Affiliations:** College of Information Technology, Shanghai Ocean University, Shanghai, 201306, China; Key Laboratory of Fisheries Information Ministry of Agriculture, Shanghai, 201306, China; College of Information Technology, Shanghai Ocean University, Shanghai, 201306, China; Key Laboratory of Fisheries Information Ministry of Agriculture, Shanghai, 201306, China; College of Information Technology, Shanghai Ocean University, Shanghai, 201306, China; Key Laboratory of Fisheries Information Ministry of Agriculture, Shanghai, 201306, China; College of Information Technology, Shanghai Ocean University, Shanghai, 201306, China; Key Laboratory of Fisheries Information Ministry of Agriculture, Shanghai, 201306, China

## Abstract

Predicting the sensitivity of tumours to specific anti-cancer treatments is a crucial area of research in precision medicine. However, it is a great challenge to propose a prediction method with high predictive accuracy and interpretability. In this study, a multi-branch interpretable deep learning model was developed to predict drug sensitivity based on multi-omics data. Three subnetworks, including a visual neural subnetwork embedded with the hierarchical structure of biological processes based on the genomic data, a subnetwork built upon the transformer encoder architecture based on methylation data, and a conventional artificial neural subnetwork based on the chemical information of drugs, were unified to predict the drug sensitivity. Cross-validation demonstrates that our model achieves the highest predictive performance over existing models. Meanwhile, the proposed model offers biologically meaningful interpretability by revealing key genetic and chemical signatures that influence drug response, identifying biological processes highly relevant to the mechanism of drug action, and capturing CpG sites on genes closely related to cancer and its treatment. In conclusion, the proposed model provides a rational therapeutic strategy for the personalized treatment of cancer patients, enabling accurate prediction of drug sensitivity and guiding clinicians toward more effective individualized regimens.

